# Pre-operative prognostic nutrition index and post-operative pneumonia in aneurysmal subarachnoid hemorrhage patients

**DOI:** 10.3389/fneur.2023.1045929

**Published:** 2023-04-28

**Authors:** Manman Xu, Liang Zhang, Juan Wang, Longyang Cheng, Chunlei Chen, Shaoya Li, Haibin Dai, Penglai Zhao, Chunhua Hang

**Affiliations:** ^1^Department of Neurosurgery, Nanjing Drum Tower Hospital, The Affiliated Hospital of Nanjing University Medical School, Nanjing, China; ^2^Department of Radiology, Nanjing Drum Tower Hospital, The Affiliated Hospital of Nanjing University Medical School, Nanjing, China

**Keywords:** prognostic nutrition index, aneurysmal subarachnoid hemorrhage, post-operative pneumonia, restricted cubic spline, net reclassification improvement, integrated discrimination improvement

## Abstract

**Background and objective:**

Post-operative pneumonia (POP), a common complication, may be associated with prolonged hospitalization and long-term mortality in aneurysmal subarachnoid hemorrhage (aSAH) patients. This study aimed to explore the association between pre-operative prognostic nutrition index (PNI) and POP in aSAH patients.

**Methods:**

A total of 280 aSAH patients were enrolled from Nanjing Drum Tower Hospital. PNI was calculated as follows: [10 × albumin(gr/dl)] + [0.005 × absolute pre-operative lymphocyte count (per mm^3^)]. We utilized multivariate analyses, restricted cubic spline, net reclassification improvement (NRI), and integrated discrimination improvement (IDI) to elucidate the role of PNI in POP.

**Results:**

Pre-operative PNI levels in the POP group were higher, compared with the non-POP group (41.0 [39.0, 45.4] vs. 44.4 [40.5, 47.3], *P* = 0.001). When we included PNI as a categorical variable in the multivariate analysis, the levels of PNI were associated with POP (odds ratio, 0.433; 95% confidence interval, 0.253–0.743; *P*=0.002). In addition, when we included PNI as a continuous variable in the multivariate analysis, the PNI levels were also associated with POP (odds ratio, 0.942; 95% confidence interval, 0.892–0.994; *P* = 0.028). The level of albumin was also a predictor of the occurrence of POP, with a lower diagnostic power than PNI [AUC: 0.611 (95% confidence interval, 0.549–0.682; *P* = 0.001) for PNI vs. 0.584 (95% confidence interval, 0.517–0.650; *P* = 0.017) for albumin]. Multivariable-adjusted spline regression indicated a linear dose–response association between PNI and POP in aSAH participants (*P* for linearity = 0.027; *P* for non-linearity = 0.130). Reclassification assessed by IDI and NRI was significantly improved with the addition of PNI to the conventional model of POP in aSAH patients (NRI: 0.322 [0.089–0.555], *P* = 0.007; IDI: 0.016 [0.001–0.031], *P* = 0.040).

**Conclusion:**

The lower levels of pre-operative PNI may be associated with the higher incidence of POP in aSAH patients. Neurosurgeons are supposed to pay more attention to pre-operative nutrition status in aSAH patients.

## Introduction

Aneurysmal subarachnoid hemorrhage (aSAH) is one of the severe neurosurgical diseases, which leads to a grave health burden (1–4). A fraction of aSAH patients may develop several common complications, such as post-operative pneumonia (POP), even if they undergo standardized operational treatment (5–8). According to previous studies, POP tends to be associated with prolonged hospitalization and long-term mortality in aSAH patients and may further aggravate family burden (6, 9). To a large extent, in the perioperative management of aSAH patients, early detection and active prevention of POP are therefore crucial.

Nutritional status is important for the management of surgical patients or patients with cerebrovascular diseases (10, 11). The prognostic nutritional index (PNI) is a novel index, which consists of the levels of albumin and lymphocyte counts and could represent the status of nutritional immune inflammation (12, 13). Ma M et al. found that the lower PNI might be associated with the worse New York Heart Association classification of coronary heart disease patients (12). Based on the analysis of the data derived from Multiparameter Intelligent Monitoring in Intensive Care, the PNI was an independent predictor of 30-day, 90-day, and 1-year mortality of critically ill patients with stroke (13). However, few studies have elucidated the role of pre-operative PNI in POP. For this reason, this study aims to explore the association between pre-operative PNI and POP in patients with aSAH.

## Methods

### Participants

In this retrospective observational study, we enrolled aSAH patients who underwent an operation in the Department of Neurosurgery, Nanjing Drum Tower Hospital, the Affiliated Hospital of Nanjing University Medical School, from June 2018 to May 2022. This study was approved by the institutional review board of Nanjing Drum Tower Hospital. All patients were managed according to the American Heart Association/American Stroke Association guidelines (14). The institutional review board waived the requirement for informed consent since no intervention was performed and no personally identifiable information appeared.

The inclusion criteria were as follows:

(1) Admission within 24 h after onset of aSAH.
(2) SAH was diagnosed by computed tomography (CT), and the diagnosis of an aneurysm was achieved by digital subtraction angiography.
(3) Undergoing an operation.
(4) Age 18 years or above.

The exclusion criteria were as follows:

(1) SAH due to other possible causes.
(2) Pre-operative pneumonia, inflammatory/infectious disease, or autoimmune diseases.
(3) Antibiotics before hospitalization.
(4) Hospitalization ≤ 72 h.
(5) Incomplete pre-operative data.

**Figure 1:**
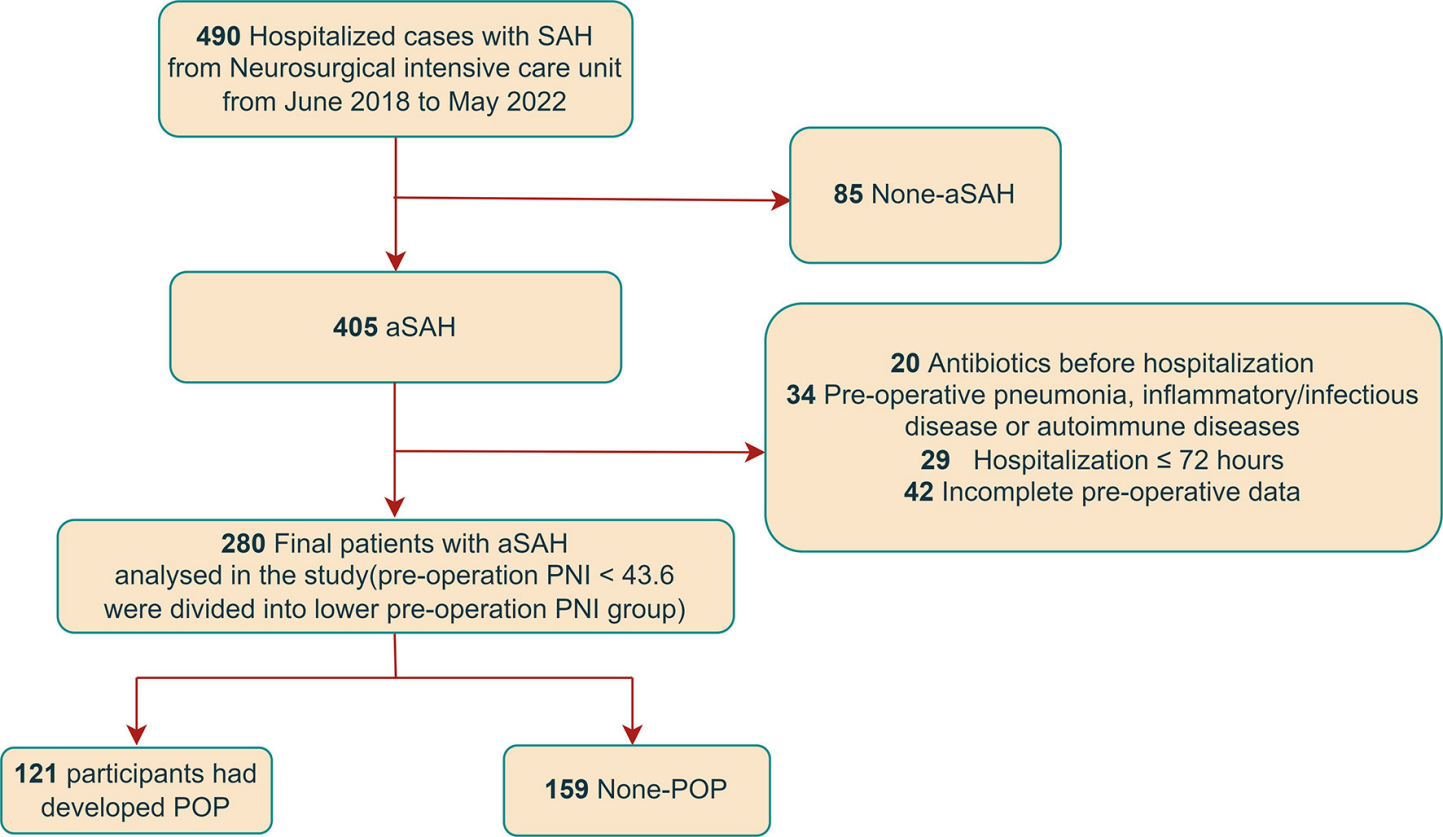
Flowchart.

The details of the enrollment are shown in Figure 1.

### Clinical data

The clinical data we collected included age, gender, hypertension, diabetes mellitus, atrial fibrillation, alcohol abuse (never, currently, and formerly), tobacco use (never, currently, and formerly), aneurysm location (anterior circulation and posterior circulation), the Hunt–Hess scale, the World Federation of Neurological Surgeons (WFNS) scale, the mFisher scale, systolic blood pressure, diastolic blood pressure, heart rate, and laboratory data (white blood cell counts, lymphocyte counts, albumin, fast blood glucose, uric acid, and C-reactive protein). Laboratory data were measured before operation. PNI was calculated as follows: [10 × albumin(gr/dl)] + [0.005 × absolute pre-operative lymphocyte count (per mm^3^)] (12, 15).

POP was diagnosed by two researchers who were blinded to other clinical and laboratory findings during hospitalization. Patients who developed lower respiratory tract infection after the operation received a diagnosis of POP according to the modified Centers for Disease Control and Prevention criteria. In the current study, either possible or confirmed pneumonia as determined by these criteria was included as pneumonia (6, 16, 17).

### Statistical analysis

Data were analyzed using SPSS version 22.0 (SPSS Inc., Chicago, IL) and R version 4.1.0 software (http://www.R-project.org/). We presented baseline characteristics of the study participants as mean ± standard deviation or median (interquartile range) for continuous variables and as frequency (percentage) for categorical variables. Univariate analysis was performed using Student's *t*-test or the Mann–Whitney U-test for continuous variables and the Fisher exact test or χ^2^ test for categorical variables, where appropriate. To confirm the association between PNI and POP, we also constructed multivariate logistic regression, including PNI as a continuous or categorical variable, respectively. The multivariate analysis was adjusted for the characteristics with a *P*-value of < 0.1 in univariate analysis. Restricted cubic splines (RCS) were performed to explore the shapes of the associations between pre-operative PNI and POP with five knots (at the 10th, 30th, 50th, 70th, and 90th percentiles). Moreover, we then utilized two statistical indexes, net reclassification improvement (NRI) and integrated discrimination improvement (IDI), to assess improvement in model performance to predict POP, by adding PNI. A *P*-value of <0.05 was considered to be statistically significant.

## Results

From June 2018 to May 2022, we enrolled 280 aSAH participants who underwent an operation (Figure 1). A total of 121 (43.2%) participants had developed POP. The aSAH participants with pre-operative PNI < 43.6 were divided into the lower pre-operative PNI group, and the aSAH participants with pre-operative PNI ≥ 43.6 were divided into the higher pre-operative PNI group. Table 1 shows the characteristics of all patients referred with lower pre-operative PNI or higher pre-operative PNI. The aSAH patients with lower pre-operative PNI were older (*P* = 0.001) and have higher levels of Hunt–Hess and WFNS scales (*P* = 0.001), fast blood glucose (*P* = 0.004), C-reactive protein (*P* = 0.001), albumin (*P* = 0.001), uric acid (*P* = 0.003), lower levels of diastolic blood pressure (*P*=0.025), white blood cell counts (*P* = 0.047), and lymphocyte counts (*P* = 0.001)

**Table 1:** Characteristics of all patients referred with lower PNI or higher PNI.

**Characteristics**	**lower PNI (*n* = 139)**	**higher PNI (*n* = 141)**	***P***
Age, years	62.5 ± 10.4	57.5 ± 10.6	0.001
Male, *n* (%)	53 (38.1)	66 (46.8)	0.142
Hypertension, n (%)	85 (61.2)	85 (60.3)	0.882
Diabetes Mellitus, n (%)	11 (7.9)	6 (4.3)	0.200
Atrial Fibrillation, n (%)	1 (0.7)	1(0.7)	0.988
Alcohol Abuse, n (%)			0.574
Never	122 (87.8)	119 (84.4)	
Currently	9 (6.5)	14 (9.9)	
Ever	8 (5.8)	8 (5.7)	
Tobacco Use, *n* (%)			0.599
Never	118 (84.9)	115 (81.6)	
Currently	10 (7.2)	15 (10.6)	
Ever	11 (7.9)	11 (7.8)	
Aneurysm Location, *n* (%)			0.676
Anterior Circulation	93 (66.9)	91 (64.5)	
Posterior Circulation	46 (33.1)	50 (35.5)	
Hunt and Hess scale, score	3 (2, 4)	2 (1, 3)	0.001
WFNS scale, score	3 (2, 4)	2 (1, 3)	0.001
mFisher scale, score	3 (2, 4)	3 (2, 4)	0.148
SBP, mmHg	139.4 ± 20.9	141.0 ± 17.9	0.491
DBP, mmHg	78.8 ± 11.2	81.9 ± 11.9	0.025
Heart Rate, /min	78 (67, 85)	78 (70, 89)	0.240
White blood cell counts, × 10^9^/L	10.9 (8.6, 14.3)	11.7 (9.6, 15.1)	0.047
Lymphocyte counts, × 10^9^/L	0.80(0.60, 1.00)	1.10(0.90, 1.60)	0.001
Fast blood glucose, mmol/L	7.31 (5.97, 9.49)	6.52 (5.53, 8.01)	0.004
Albumin, g/L	35.7(33.5, 36.9)	40.9(39.3, 43.4)	0.001
C reactive protein, mmol/L	26.4 (8.40, 65.30)	17.9 (5.50, 39.40)	0.001
Uric acid, μmol/L	198 (159, 247)	232 (174.5, 329)	0.003

PNI, prognostic nutrition index; WFNS, World Federation of Neurological Surgeons; SBP, systolic blood pressure; DBP, diastolic blood pressure.

**Figure 2:**
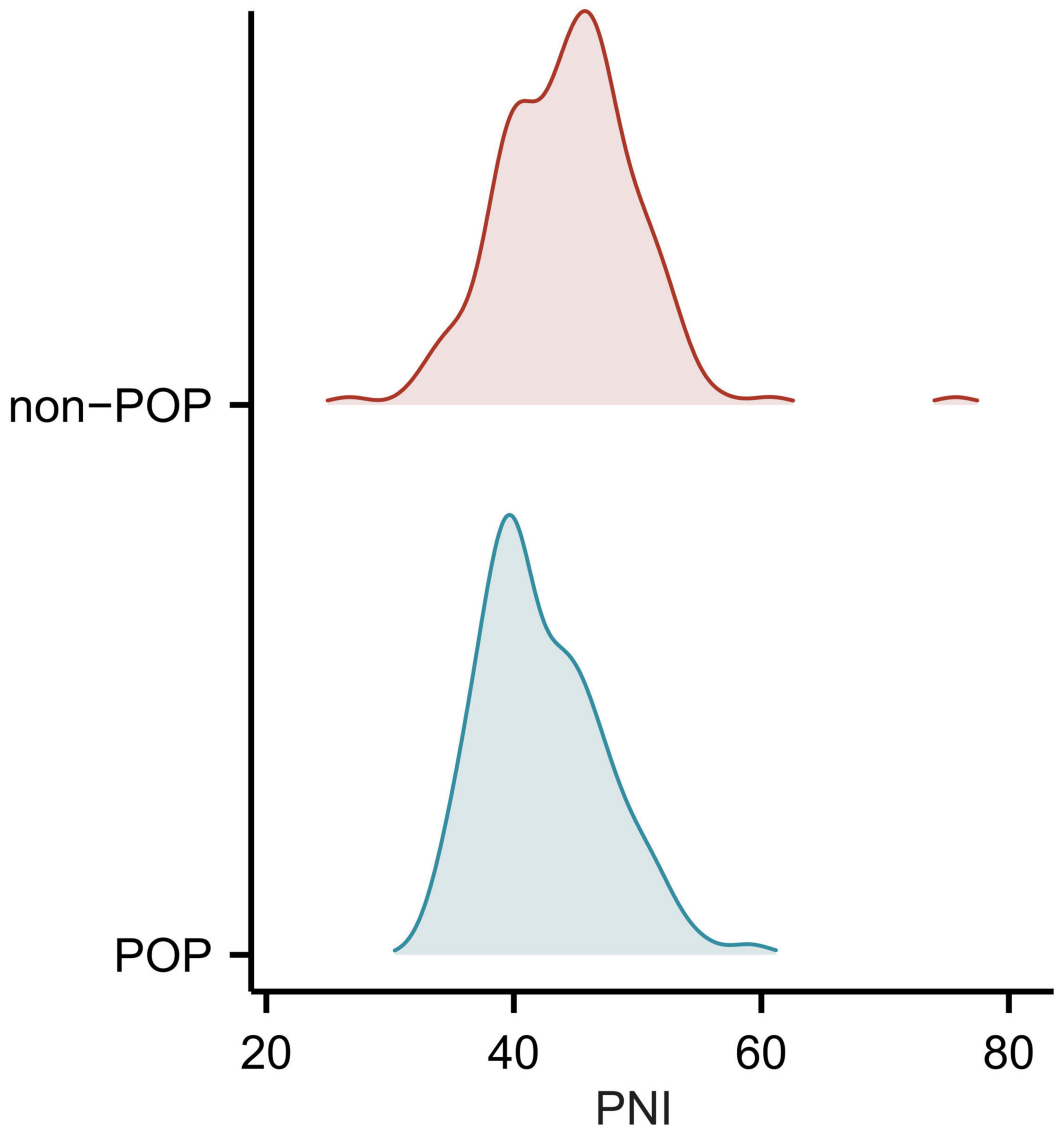
Ridge plot to present the levels of PNI in the POP group and non-POP group.

Table 2 manifests the characteristics of all patients referred with POP or without POP. The aSAH patients, who developed POP had higher levels of Hunt–Hess and WFNS scales (*P* = 0.001), mFisher scale (*P* = 0.001), white blood cell counts (*P* = 0.035), lymphocyte counts (*P* = 0.041), albumin (*P* = 0.001), fast blood glucose (*P* = 0.008), C-reactive protein (*P* = 0.035), and lower levels of PNI (*P* = 0.001). Moreover, the details of pre-operative PNI levels between the POP group and non-POP group are shown in Figure 2 (41.0 [39.0, 45.4] vs. 44.4 [40.5, 47.3], *P* = 0.001).

**Table 2:** Characteristics of all patients referred with POP.

**Characteristics**	**POP (*n* = 121)**	**Non-POP (*n* = 159)**	***P***
Age, years	61.2 ± 11.9	59.1 ± 9.8	0.111
Male, *n* (%)	56 (46.3)	63 (39.6)	0.264
Hypertension, *n* (%)	77 (63.6)	93 (58.5)	0.382
Diabetes Mellitus, *n* (%)	9 (7.4)	8 (5.0)	0.404
Atrial Fibrillation, *n* (%)	1 (0.8)	1 (0.6)	0.841
Alcohol Abuse, *n* (%)			0.695
Never	106 (87.6)	135 (84.9)	
Currently	8 (6.6)	15 (9.4)	
Ever	7 (5.8)	9 (5.7)	
Tobacco Use, *n* (%)			0.520
Never	99 (81.8)	134 (84.3)	
Currently	10 (8.3)	15 (9.4)	
Ever	12 (9.9)	10 (6.3)	
Aneurysm Location, *n* (%)			0.376
Anterior Circulation	83 (68.6)	101 (63.5)	
Posterior Circulation	38 (31.4)	58 (36.5)	
WFNS scale, score	3 (2, 4)	2 (1, 3)	0.001
mFisher scale, score	3 (2, 4)	2 (2, 4)	0.001
SBP, mmHg	140.6 ± 19.5	139.9 ± 19.5	0.775
DBP, mmHg	80.7 ± 11.4	80.1 ± 11.8	0.687
Heart Rate, /min	77.0 (70.0, 89.0)	78.0 (68.0, 85.0)	0.520
White blood cell counts, × 10^9^/L	11.7 (9.5, 16.5)	11.1 (8.8, 13.8)	0.035
Lymphocyte counts, × 10^9^/L	0.90(0.65, 1.20)	1.00(0.70, 1.30)	0.041
Fast blood glucose, mmol/L	7.31 (5.99, 9.33)	6.59 (5.52, 8.20)	0.008
C reactive protein, mmol/L	25.1 (8.2, 65.3)	20.4 (6.4, 45.0)	0.035
Albumin, g/L	36.5 (34.9, 40.0)	39.2 (36.1, 41.5)	0.001
Uric acid, μmol/L	221 (173.5, 284)	207 (162, 282)	0.532
PNI as a continuous variable	41.0 (38.9,45.4)	44.4 (40.4,47.3)	0.001
PNI as a categorical variable			0.001
Lower PNI	76 (62.8)	63 (39.6)	
Higher PNI	45 (37.2)	96 (60.4)	

POP, post-operative pneumonia; PNI, prognostic nutrition index; WFNS, World Federation of Neurological Surgeons; SBP, systolic blood pressure; DBP, diastolic blood pressure.

The multivariate analysis was adjusted for the characteristics with a *P*-value of < 0.1 in Table 2. When we included pre-operative PNI as a categorical variable in the multivariate analysis, the levels of PNI were associated with POP (odds ratio, 0.433; 95% confidence interval, 0.253–0.743; *P* = 0.002). In addition, when we included pre-operative PNI as a categorical variable in the multivariate analysis, the PNI levels were also associated with POP (odds ratio, 0.942; 95% confidence interval, 0.892–0.994; *P* = 0.028). In addition, the lower levels of albumin may be associated with a higher incidence of POP in aSAH patients. When the albumin value was adjusted in the multivariate analysis, the odds ratio for higher PNI was 0.376 (95% confidence interval, 0.187–0.757, *P* = 0.006). The level of albumin was also a predictor of the occurrence of POP, with a lower diagnostic power (*P* = 0.017, AUC = 0.584, 95% confidence interval, 0.517–0.650) than PNI (*P* = 0.001, AUC = 0.616, 95% confidence interval, 0.549–0.682). The level of lymphocyte counts did not significantly predict POP (*P* = 0.060). To avoid the possibility of inducing collinearity among the values of albumin, lymphocyte counts, and PNI, we excluded the two variables (albumin and lymphocyte counts) from the ultimate multivariate analysis. Table 3 displays the multivariate analysis for the association between pre-operative PNI and the presence of POP in aSAH patients.

**Table 3:** Multivariate analysis for the association between prognostic nutritional index and presence of POP in patients with aneurysmal subarachnoid hemorrhage.

Multivariate analysis^*^	**OR**	**95%CI**	***P***
PNI as a categorical variable			
Lower PNI	Reference	reference	
Higher PNI	0.433	0.253-0.743	0.002
PNI as a continuous variable	0.942	0.892-0.994	0.028

^*^Adjusted for Hunt and Hess scale, mFisher scale, white blood cell counts, fast blood glucose and C reactive protein.

PNI, prognostic nutrition index; POP, post-operative pneumonia; OR, odds ratio; CI, confidence interval.

**Figure 3:**
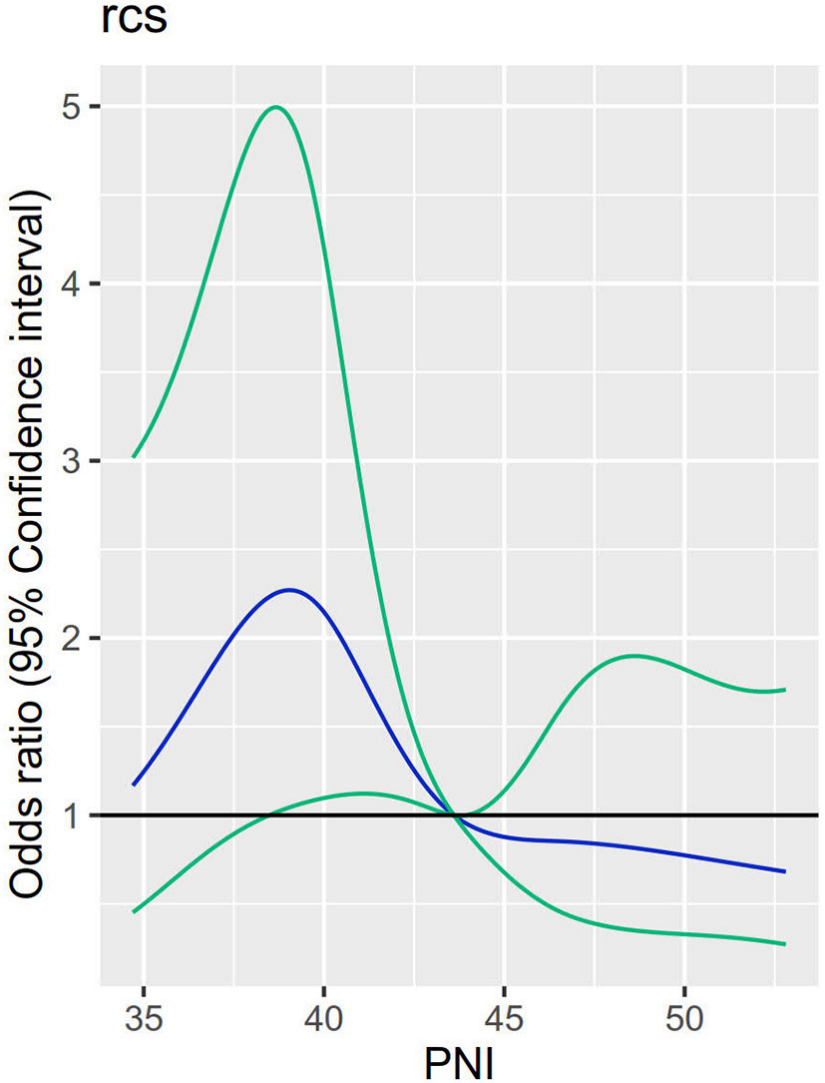
Relationship between levels of PNI and POP in aSAH patients undergoing an operation. Adjusted odds ratios and 95% confidence intervals derived from restricted cubic spline regression, with knots placed at the 10th, 30th, 50th, 70th, and 90th percentiles of PNI levels.

Figure 3 indicates a linear dose–response association between PNI before operation and the POP in aSAH participants (*P* for linearity = 0.027; *P* for non-linearity = 0.130). Reclassification assessed by IDI and NRI was significantly improved with the addition of pre-operative PNI to the conventional model of POP in aSAH patients (NRI: 0.322 [0.089–0.555], *P* = 0.007; IDI: 0.016 [0.001–0.031], *P* = 0.040; Table 4).

**Table 4:** Reclassification statistics (95% CI) for POP after the addition of prognostic nutritional index.

**Reclassification statistics**	**Estimate**	**95% CI**	***P***
Multivariate model + PNI			
NRI	0.322	0.089 - 0.555	0.007
IDI	0.016	0.001 - 0.031	0.040

Multivariate model, adjusted for Hunt and Hess scale, mFisher scale, white blood cell counts, fast blood glucose and C reactive protein.

PNI, prognostic nutrition index; POP, post-operative pneumonia; NRI, net reclassification improvement; IDI, integrated discrimination improvement.

## Discussion

We conducted this hospital-based observational study and discovered that there is a link between PNI before operation and POP in aSAH patients undergoing operation. In this study, the incidence of POP is somewhat high in our center perhaps because the aSAH patients in our center are more serious and sicker. Moreover, our careful evaluation of POP may also result in this phenomenon. The linear dose–response association between PNI before operation and the POP was confirmed by RCS analysis. In addition, pre-operative PNI could also improve the ability of reclassification for POP, which was confirmed by NRI and IDI. A previous study suggested that low caloric intake and negative nitrogen balance were related to the survival of SAH patients (18). The results of our study showed the role of nutrition in POP of aSAH patients, supplementing the important value of nutrition in patients with SAH.

### Malnutrition, PNI, and surgery

Pre-operative malnutrition plays a huge role in multiple types of surgeries. Zhang et al. have found that pre-operative malnutrition may serve as an independent risk factor for post-operative mortality in elderly Chinese individuals undergoing hip surgery (19). One study in America ascertained that pre-operative malnutrition was associated with increased mortality and adverse outcomes after pediatric cardiac surgery (20). Moreover, pre-operative malnutrition may be associated with post-operative affective disorders. Mazzola P et al. disclosed the association between pre-operative malnutrition and post-operative delirium after hip fracture surgery in older adults (21). PNI is a novel and useful index to measure the status of nutrition. A study enrolling 771 patients undergoing radical gastric cancer surgery revealed that pre-operative PNI might be an independent risk factor for the prognosis of patients with gastric cancer (22). Kanda M et al. found that in patients with pancreatic cancer, PNI could be associated with overall survival and post-operative complications, in particular pancreatic fistula (23). Another study showed that ascites and malnutrition are prediction factors for incomplete cytoreductive surgery for peritoneal carcinomatosis from gastric cancer (24).

### Malnutrition, PNI, and infection

There are also several studies that revealed the relationship between malnutrition and infection. According to a previous study, malnutrition is associated with an increased risk of developing surgical site infection after spinal surgery (25). Clinical epidemiological analysis showed that malnutrition might be able to prolong the hospitalization of patients with COVID-19 infection (26). A systematic review and meta-analysis also showed pre-operative malnutrition negatively correlated with post-operative infection after total joint arthroplasty (27). Moreover, one recent research has found a relationship between PNI and new-onset pneumonia in peritoneal dialysis patients (28). The present study demonstrated that PNI and level of albumin were associated with the risk of POP, which was partly in accordance with previous research (7).

### Malnutrition, PNI, and cerebrovascular diseases

It is also vital to pay attention to the nutritional status of patients with cerebrovascular diseases.

The relationship between nutrition and rehabilitation in stroke patients is relatively recognized (29, 30). Yuan K et al. found that malnutrition might be associated with long-term mortality in older adults with ischemic stroke (31). From the results of another research, PNI was an independent predicting factor of 30-day, 90-day, and 1-year mortality of critically ill patients with stroke (13). Nutritional risk assessment may be related to outcomes in SAH patients hospitalized in a neurointensive critical care unit (32).

### Strengths and limitations

Nevertheless, the study on PNI and POP in aSAH patients is relatively rare. To the best of our knowledge, our study is the first to investigate the link between pre-operative PNI and POP in aSAH undergoing operation, further enhancing the clinical role of PNI in surgery, infection, and cerebrovascular diseases.

This study has several flaws and limitations. First, it is important to consider the relatively small size of the sample in this study. We enrolled aSAH patients undergoing an operation in only one center, so the conclusion may not be applied to patients at other centers. Second, dynamic observations of nutritional status will be needed in future studies to further explore the relationship between nutritional status and POP in aSAH patients. Third, several aSAH patients undergoing operation left the hospital earlier. Therefore, we excluded these patients because we did not know whether these patients developed POP, which may lead to bias. Last, the observational design makes it difficult to establish a causal association between nutritional status and POP in aSAH patients undergoing an operation. Further longitudinal investigations with precision design are warranted in the future.

### Summary

In summary, this observational study revealed that pre-operative PNI might be associated with the occurrence of POP in patients with aSAH. The neurosurgeon is supposed to pay attention to the nutritional status of aSAH patients. Future research could focus on whether improving nutritional status is able to reduce the occurrence of POP in aSAH patients undergoing an operation.

## Data availability statement

The data that support the findings of this study are available from the corresponding author upon reasonable request.

## Ethics statement

The studies involving human participants were reviewed and approved by the Institutional Review Board of Nanjing Drum Tower Hospital. The Ethics Committee waived the requirement of written informed consent for participation.

## Author contributions

MX designed the study, interpreted the results, and wrote the manuscript. LZ and CH collected the study data and interpreted the results. JW, LC, CC, SL, and HD performed data analysis and revised the manuscript. PZ designed and revised the manuscript. All authors contributed to the article and approved the submitted version.

## References

[1] Spantler D Molnar T Simon D Berki T Buki A Schwarcz A. Biomarker associations in delayed cerebral ischemia after aneurysmal subarachnoid hemorrhage. Int J Mol Sci. (2022) 23:8789. 10.3390/ijms23158789 35955921 PMC9369444

[2] Hu P Li Y Liu Y Guo G Gao X Su Z. Comparison of conventional logistic regression and machine learning methods for predicting delayed cerebral ischemia after aneurysmal subarachnoid hemorrhage: a multicentric observational cohort study. Front Aging Neurosci. (2022) 14:857521. 10.3389/fnagi.2022.857521 35783143 PMC9247265

[3] Lu H Xue G Li S Mu Y Xu Y Hong B. An accurate prognostic prediction for aneurysmal subarachnoid hemorrhage dedicated to patients after endovascular treatment. Ther Adv Neurol Disord. (2022) 15:17562864221099473. 10.1177/17562864221099473 35677817 PMC9168851

[4] Liu F Bao Y Qiu B Mao J Liao X Huang H. Identification of novel cerebrospinal fluid biomarkers for cognitive decline in aneurysmal subarachnoid hemorrhage: a proteomic approach. Front Cell Neurosci. (2022) 16:861425. 10.3389/fncel.2022.861425 35602555 PMC9120969

[5] Yuan K Li R Zhao Y Wang K Lin F Lu J. Pre-operative predictors for post-operative pneumonia in aneurysmal subarachnoid hemorrhage after surgical clipping and endovascular coiling: a single-center retrospective study. Front Neurol. (2022) 13:893516. 10.3389/fneur.2022.893516 35812098 PMC9263125

[6] Wang R Zhang J He M Xu J. A novel risk score for predicting hospital acquired pneumonia in aneurysmal subarachnoid hemorrhage patients. Int Immunopharmacol. (2022) 108:108845. 10.1016/j.intimp.2022.108845 35609376

[7] Zhang X Zhang S Wang C Liu R Li A. High neutrophil-to-albumin ratio predicts postoperative pneumonia in aneurysmal subarachnoid hemorrhage. Front Neurol. (2022) 13:840858. 10.3389/fneur.2022.840858 35463142 PMC9021997

[8] Meng N Ye Z Liu Y Qin C Su Y. Impact of the 'weekend effect' on hospital-acquired pneumonia after aneurysmal subarachnoid hemorrhage. Postgrad Med. (2021) 133:974–8. 10.1080/00325481.2021.1959936 34323649

[9] Li R Lin F Chen Y Lu J Han H Yan D. In-hospital complication-related risk factors for discharge and 90-day outcomes in patients with aneurysmal subarachnoid hemorrhage after surgical clipping and endovascular coiling: a propensity score-matched analysis. J Neurosurg. (2021) 137:381–92. 10.3171/2021.10.JNS211484 34972088

[10] Hamamah S Hajnal A Covasa M. Impact of nutrition, microbiota transplant and weight loss surgery on dopaminergic alterations in Parkinson's disease and obesity. Int J Mol Sci. (2022) 23:7503. 10.3390/ijms23147503 35886849 PMC9319073

[11] Yoshimura Y. Recent advances in clinical nutrition in stroke rehabilitation. Nutrients. (2022) 14:1130. 10.3390/nu14061130 35334787 PMC8953342

[12] Ma M Liu Y Liu F Li Z Cheng Q Liu Z. Relationship between prognostic nutrition index and New York heart association classification in patients with coronary heart disease: a RCSCD-TCM study. J Inflamm Res. (2022) 15:4303–14. 10.2147/JIR.S371045 35923911 PMC9342891

[13] Liu Y Yang X Kadasah S Peng C. Clinical value of the prognostic nutrition index in the assessment of prognosis in critically Ill patients with stroke: a retrospective analysis. Comput Math Methods Med. (2022) 2022:4889920. 10.1155/2022/4889920 35586667 PMC9110188

[14] Connolly ES Jr Rabinstein AA Carhuapoma JR Derdeyn CP Dion J Higashida RT. American heart association stroke council; council on cardiovascular radiology and intervention; council on cardiovascular nursing; council on cardiovascular surgery and anesthesia; council on clinical cardiology. Guidelines for the management of aneurysmal subarachnoid hemorrhage: a guideline for healthcare professionals from the American Heart Association/american Stroke Association. Stroke. (2012) 43:1711–37. 10.1161/STR.0b013e3182587839 22556195

[15] Cadwell JB Afonso AM Shahrokni A. Prognostic nutritional index (PNI), independent of frailty is associated with six-month postoperative mortality. J Geriatr Oncol. (2020) 11:880–4. 10.1016/j.jgo.2020.03.013 32253157 PMC8311543

[16] Ding CY Peng L Lin YX Yu LH Wang DL Kang DZ. Elevated lactate dehydrogenase level predicts postoperative pneumonia in patients with aneurysmal subarachnoid hemorrhage. World Neurosurg. (2019) 129:e821–30. 10.1016/j.wneu.2019.06.041 31203058

[17] Smith CJ Kishore AK Vail A Chamorro A Garau J Hopkins SJ. Diagnosis of stroke-associated pneumonia: recommendations from the pneumonia in stroke consensus group. Stroke. (2015) 46:2335–40. 10.1161/STROKEAHA.115.009617 26111886

[18] Badjatia N Ryan A Choi HA Parikh GY Jiang X Day AG. Relationship between nutrition intake and outcome after subarachnoid hemorrhage: results from the international nutritional survey. J Intensive Care Med. (2021) 36:1141–8. 10.1177/0885066620966957 34519558

[19] Feng L Chen W Ping P Ma T Li Y Xu L. Preoperative malnutrition as an independent risk factor for the postoperative mortality in elderly Chinese individuals undergoing hip surgery: a single-center observational study. Ther Adv Chronic Dis. (2022) 13:20406223221102739. 10.1177/20406223221102739 35782344 PMC9243382

[20] Ross F Latham G Joffe D Richards M Geiduschek J Eisses M. Preoperative malnutrition is associated with increased mortality and adverse outcomes after paediatric cardiac surgery. Cardiol Young. (2017) 27:1716–25. 10.1017/S1047951117001068 28625194 PMC5908464

[21] Mazzola P Ward L Zazzetta S Broggini V Anzuini A Valcarcel B. Association between preoperative malnutrition and postoperative delirium after hip fracture surgery in older adults. J Am Geriatr Soc. (2017) 65:1222–8. 10.1111/jgs.14764 28263371 PMC6555399

[22] Xu Z Chen X Yuan J Wang C An J Ma X. Correlations of preoperative systematic immuno-inflammatory index and prognostic nutrition index with a prognosis of patients after radical gastric cancer surgery. Surgery. (2022) 172:150–9. 10.1016/j.surg.2022.01.006 35168816

[23] Kanda M Fujii T Kodera Y Nagai S Takeda S Nakao A. Nutritional predictors of postoperative outcome in pancreatic cancer. Br J Surg. (2011) 98:268–74. 10.1002/bjs.7305 20960457

[24] Benizri EI Bereder JM Rahili A Bernard JL Benchimol D. Ascites and malnutrition are predictive factors for incomplete cytoreductive surgery for peritoneal carcinomatosis from gastric cancer. Am J Surg. (2013) 205:668–73. 10.1016/j.amjsurg.2012.06.009 23369310

[25] Tsantes AG Papadopoulos DV Lytras T Tsantes AE Mavrogenis AF Koulouvaris P. Association of malnutrition with surgical site infection following spinal surgery: systematic review and meta-analysis. J Hosp Infect. (2020) 104:111–9. 10.1016/j.jhin.2019.09.015 31562915

[26] Shang S Huang Y Zhan X Peng F Wang X Wen Y. The relationship between the prognostic nutritional index and new-onset pneumonia in peritoneal dialysis patients. Int Urol Nephrol. (2022) 54:3017–24. 10.1007/s11255-022-03233-1 35701571 PMC9197727

[27] Gu A Malahias MA Strigelli V Nocon AA Sculco TP Sculco PK. Preoperative malnutrition negatively correlates with postoperative wound complications and infection after total joint arthroplasty: a systematic review and meta-analysis. J Arthroplasty. (2019) 34:1013–24. 10.1016/j.arth.2019.01.005 30745081

[28] Yu Y Ye J Chen M Jiang C Lin W Lu Y. Malnutrition prolongs the hospitalization of patients with COVID-19 infection: a clinical epidemiological analysis. J Nutr Health Aging. (2021) 25:369–73. 10.1007/s12603-020-1541-y 33575730 PMC7709472

[29] Sabbouh T Torbey MT. Malnutrition in stroke patients: risk factors, assessment, and management. Neurocrit Care. (2018) 29:374–84. 10.1007/s12028-017-0436-1 28799021 PMC5809242

[30] Aquilani R Sessarego P Iadarola P Barbieri A Boschi F. Nutrition for brain recovery after ischemic stroke: an added value to rehabilitation. Nutr Clin Pract. (2011) 26:339–45. 10.1177/0884533611405793 21586419

[31] Yuan K Zhu S Wang H Chen J Zhang X Xu P. Association between malnutrition and long-term mortality in older adults with ischemic stroke. Clin Nutr. (2021) 40:2535–42. 10.1016/j.clnu.2021.04.018 33932800

[32] Carvalho MR Berto BSD Rodrigues AM Prudente LOB Moura ELB. Nutritional assessment of patients with aneurysmal subarachnoid hemorrhage using the modified “Nutrition Risk in the Critically ill” score, and its association with outcomes. Nutr Hosp. (2022) 39:709–15. 10.20960/nh.04093 35916136

